# Spatiotemporal NO/O_2_-releasing cascade nanozyme microneedles enhance diabetic infected wound healing by modulating the immune microenvironment

**DOI:** 10.7150/thno.123827

**Published:** 2026-01-01

**Authors:** Hanqing Zhao, Wenjun Shao, Man Jiang, Fangyuan Chen, Yingying Li, Xiaoyu Zhang, Tao Ma, Minglong Chen, Baohong Sun, Zekun Wang, Chuanbin Wu, Qingqing Wang

**Affiliations:** 1 School of Pharmacy, Bengbu Medical University, Bengbu, Anhui, 233030, China.; 2 Anhui Engineering Technology Research Center of Biochemical Pharmaceutical, Bengbu Medical University, Bengbu, Anhui, 233030, China.; 3 Department of Pharmacy, The First Affiliated Hospital of the University of Science and Technology of China, and State Key Laboratory of Precision and Intelligent Chemistry, University of Science and Technology of China, Hefei, Anhui, 230026, China.; 4 Interdisciplinary Eye Research Institute (EYE-X Institute), Bengbu Medical University, Bengbu, Anhui, 233030, China.; 5 College of Pharmacy, Jinan University, Guangzhou 511443, China.

**Keywords:** cascade reaction, nanozyme, nitric oxide, dissolvable microneedles, diabetes biofilm wounds

## Abstract

**Rationale:** Chronic diabetic wounds present significant therapeutic challenges due to biofilm resistance and dysregulated metabolism of glucose, reactive oxygen species (ROS), and nitric oxide (NO), which collectively exacerbate immunosuppression and impair tissue repair.

**Methods:** This study developed a glucose-driven cascade nanozyme-loaded dissolvable microneedle system (PPLG@MN) that enables spatiotemporal regulation of the wound microenvironment through a closed-loop mechanism involving nutrient deprivation, gas modulation, and immune reprogramming. The system consists of porous Prussian blue nanozymes (PPB) loaded with glucose oxidase (GOx) and L-arginine (L-arg), which is precisely delivered into the tissue via microneedles.

**Results:** PPLG@MN initiates a self-sustaining therapeutic cycle within the biofilm microenvironment, in which GOx catalyzes the oxidation of glucose to generate hydrogen peroxide (H_2_O_2_), inducing bacterial starvation. Subsequently, H_2_O_2_ reacts with PPB and L-arg to release oxygen (O_2_) and biofilm-disrupting NO, thereby alleviating local hypoxia and enhancing antibacterial efficacy. Furthermore, the synergistic action of O_2_ and NO reprograms macrophages toward an anti-inflammatory M2 phenotype (approximately 30-fold increase compared to the model group), effectively resolving inflammation and promoting angiogenesis. *In vivo* studies confirmed that the system achieved > 99.9% biofilm eradication efficiency and accelerated wound healing by 25.6% compared to the model group.

**Conclusion:** This nanoplatform offers a clinically translatable therapeutic strategy for biofilm-associated diabetic wounds by synergistically combining gas therapy and immune reprogramming.

## Introduction

In recent years, diabetes mellitus has shown an increasing prevalence globally and is occurring more frequently among younger populations, with patient numbers projected to exceed 10% worldwide by 2030 1-3. Hyperglycemic wounds provide a favorable environment for bacterial colonization, and elevated glucose levels disrupt endothelial nitric oxide synthase activity, leading to reduced nitric oxide (NO) production and impaired angiogenesis 4. Nitric oxide can activate neutrophils and macrophages, and facilitate the synthesis of collagen, significantly accelerating the regeneration of traumatized tissues 5. Consequently, diabetic wounds often heal poorly and, in severe cases, may require amputation, posing a significant threat to human health 6,7. The wound healing process involves multiple coordinated stages, with the complex microenvironment further complicating treatment 8. Effective healing requires the targeted regulation of signaling molecules to repair tissue damage 9-11. However, traditional treatments, including debridement, wound dressings, and antibiotics, often prove insufficient. The nutrient-rich environment of these wounds supports bacterial proliferation and biofilm formation, which are notoriously difficult to eradicate and thus perpetuate chronic infections at the wound site 12,13. These infections result in the excessive release of inflammatory mediators, stalling the healing process during the inflammatory phase and inhibiting cell migration and proliferation 14,15. This further leads to reduced angiogenic activity and difficulty in entering the proliferative phase due to persistent infection and inflammatory stimuli 16,17. Collectively, these interconnected factors make diabetic wound healing particularly challenging, emphasizing the urgent need for an integrated therapeutic approach to promote optimal healing outcomes.

Eliminating bacterial infections is critical for promoting chronic wound healing. Currently, clinical management of wound biofilms commonly involves debridement combined with antibiotics 18. However, debridement often causes significant pain, reducing patient compliance, while excessive antibiotic use contributes to bacterial resistance and the emergence of "super bacteria" 19,20. Therefore, new strategies that simultaneously eradicate bacteria and prevent biofilm recurrence are urgently required. Gas therapy is widely recognized for its efficacy in treating bacterial infections. Gases such as NO, carbon monoxide (CO) and hydrogen sulfide (H_2_S) are commonly utilized 21-23. NO is particularly advantageous due to its potent, broad-spectrum antimicrobial properties, inducing irreversible nitrosative and oxidative damage to microbial proteins, DNA, and membranes, alongside its multiple tissue-repairing capabilities 24,25. However, direct gaseous application of NO faces limitations, including difficult storage and uncontrolled release. L-arginine (L-arg), an essential amino acid in the human body, can react with reactive oxygen species (ROS) to continuously generate NO, making it a more ideal NO donor 26. Nevertheless, typical NO donors are restricted by short physiological half-lives, low bioavailability, and systemic side effects, necessitating carrier-mediated controlled and sustained NO delivery 27.

Nano-delivery systems offer targeted delivery, extended release, enhanced bioavailability, and reduced systemic toxicity, gaining increasing attention in biomedical applications 28-30. Among these systems, nanozymes integrate the unique properties of nanomaterials with enzymatic functions. Prussian blue (PB) nanozymes exhibit catalase (CAT) activity and anti-inflammatory properties, and their porous structures can be designed to enhance drug delivery efficiency 31-33. According to this concept, porous PB nanozymes can deliver L-arg and generate NO via its reaction with hydrogen peroxide (H_2_O_2_) in the biofilm microenvironment, thereby killing bacteria and disrupting the biofilm. However, the bactericidal effect of NO is concentration-dependent, and the H_2_O_2_ concentration in the wound microenvironment is insufficient to directly eliminate bacteria and continuously generate NO 34. Additionally, hyperglycemia-induced angiogenic suppression further impedes wound healing 35. Glucose oxidase (GOx), a natural enzyme, consumes glucose to generate gluconic acid and H_2_O_2_ in the presence of oxygen, the latter of which promotes the production of high concentrations of NO 36. Thus, combining GOx, L-arg, and PB forms a cascade system capable of glucose depletion, pH normalization, NO generation, and biofilm disruption. Nevertheless, physical barriers like biofilms in chronic diabetic wounds hinder effective drug delivery 37. Dissolvable microneedles (DMN), a novel transdermal delivery system combining advantages of injections and dressings, penetrate biofilms directly, significantly enhancing therapeutic efficacy.

Although several studies have explored glucose- or H_2_O_2_-responsive nanoplatforms incorporating GOx with NO donors, these systems typically suffer from limited catalytic depth within biofilms, inability to couple NO release with dynamic metabolic transitions in diabetic wounds, and dependence on externally added NO donors that lack microenvironment adaptivity. Previous reports also primarily relied on nanoparticles or hydrogels, which cannot actively breach biofilm barriers or coordinate multistage wound repair 30,38. In contrast, the present PPLG@MN platform differentiates itself by integrating (i) biofilm-penetrating dissolvable microneedles, (ii) a self-sustaining NO/O_2_ cascade driven solely by endogenous glucose and oxygen, (iii) porous PB nanozymes enabling stable catalytic amplification, and (iv) dynamic spatiotemporal regulation of NO that sequentially supports antibacterial activity, immunomodulation, and angiogenesis. Such multilayered adaptivity and DMN-enabled direct microenvironment access have not been reported in existing glucose/H_2_O_2_-responsive nanozyme systems or in GOx + NO donor constructs.

**Scheme 1:**
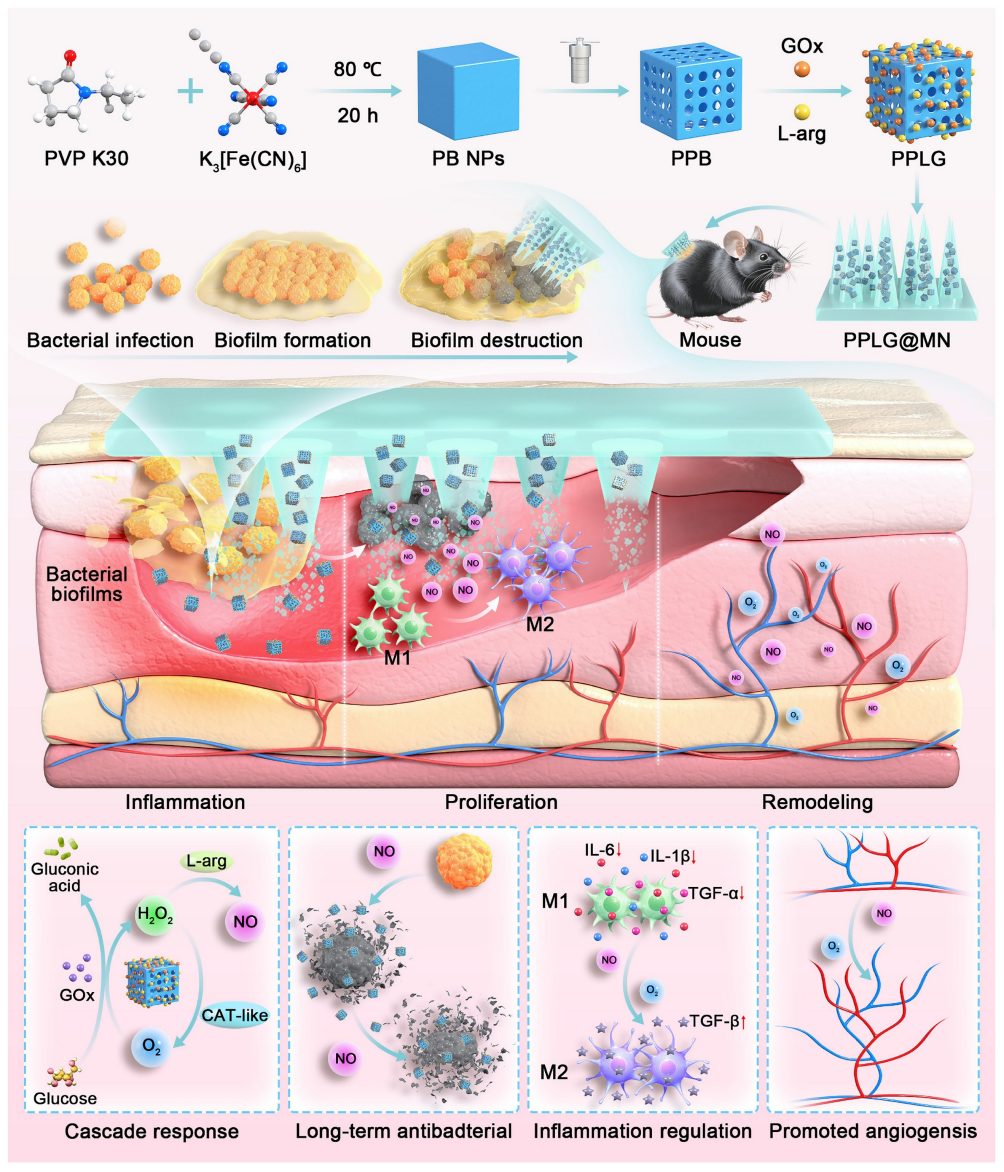
Schematic diagram of PPLG@MN for the treatment of diabetic biofilm wounds infected with *S. aureus*. The figure illustrates the synthesis of PPLG, the preparation of PPLG@MN, and the *in vivo* wound healing effects in diabetic mice. The cascade reaction involves the consumption of excess glucose to release H_2_O_2_-NO, which collectively contributes to bacterial inhibition, inflammation reduction, and the promotion of angiogenesis.

In this study, we developed a DMN carrying a cascade nanozyme system (PPLG@MN) designed specifically for diabetic biofilm wounds. As shown in Scheme 1, we synthesized a porous Prussian blue nanozyme (PPB), subsequently loading L-arg and GOx to form a cascade nanosystem. This configuration enables controlled, sustained NO release, addressing the critical limitation of uncontrolled NO delivery inherent in gas therapy. Upon application, PPLG@MN mechanically disrupts biofilms, facilitating cascade nanozyme penetration to lesion sites, initiating sequential reactions: glucose consumption, elevated NO production, and synergistic physicochemical antibacterial effects. The nanozyme initiates a cascade reaction, consuming excess glucose, producing higher concentrations of NO, and exerting a synergistic physical and chemical effect to kill bacteria and disrupt biofilms. Additionally, the cascade reaction reduces the excessive secretion of inflammatory factors. As the reaction progresses, the wound microenvironment becomes slightly acidic, resembling normal skin, due to the continuous generation of gluconic acid. The cascade reaction gradually slows, leading to a decrease in NO production, which ultimately promotes macrophage polarization toward the M2 phenotype and enhances endothelial cell migration and angiogenesis, thereby accelerating wound healing. The dynamic spatiotemporal regulation of NO by the PPLG system, demonstrated by *in vitro* and *in vivo* experiments, represents an innovative therapeutic strategy for diabetic biofilm wound management.

## Materials and Methods

### Reagents and animals

Potassium ferricyanide, 1-naphthol, chitosan and GOx were obtained from McLean Limited (Shanghai, China). Streptozotocin (STZ) was purchased from Sigma. The hydrogen peroxide content detection kit, reactive oxygen species detection kit, and cell activity and cytotoxicity detection kit were all purchased from Beyotime Biotechnology (Shanghai, China). L-arginine (L-arg) was purchased from Aladdin Limited (Shanghai, China). Kollidon^®^90 and Kollidon^®^30 were both purchased from BASF (Germany). Eight-week-old male C57BL/6J mice were provided by Hangzhou Ziyuan Experimental Animal Technology *Co., Ltd*. (Hangzhou, China). All animal studies were approved by the Animal Ethics Committee of Bengbu Medical University (Bengbu, China). Animal experiments were approved in accordance with the Animal Ethics Committee of Anhui Province (No. 170 [2023]).

### Preparation of the porous Prussian blue nanozyme

PVP K30 (3 g), K_3_FeC_6_N_6_ (113.14 mg), HCl (40 mL) were weighed and added into a 100 mL flask and stirred for 30 min, after which the mixture was heated to 80 ℃ and placed in the flask for 20 h. After cooling, the supernatant was centrifuged and the precipitate was washed and repeated three times to obtain PB NPs. A certain amount of PB NPs and PVP K30 was dissolved in HCl solution and stirred for 120 min on a magnetic stirrer. The solution was then transferred to a reactor for a high-temperature reaction for 4 h, centrifuged, and the supernatant was discarded. The precipitate was washed and then transferred to a vacuum drying oven and dried for 12 h. The PPB product was finally dried for 12 h. After cooling, centrifuged and discarded the supernatant and washed the precipitation, and the precipitate was then dried for 12 h, and obtained PPB product.

### Preparation of cascade nanozymes

Appropriate amount of PPB powder was dissolved in ultrapure water, GOx and L-arg powder were added to form a 2:1 feeding ratio respectively, and the further formed solution was stirred under a magnetic stirrer for 24 h. The product was washed with ultrapure water for three times to obtain the PPLG, and freeze-dried for 48 h to obtain the dried final product. The adsorption capacity of GOx was determined using the MicroBCA method, and the loading capacity of L-Arg was quantified with the Sakaguchi reagent.

### Preparation of dissolvable microneedles (MN)

PPLG (40 mg) and HA (200 mg) were dissolved in deionized water (1 mL) to form a homogeneous needle stock solution. This solution was added to a PDMS mould, centrifuged (3500 rpm, 25 ℃, 4 min), scraped to remove excess, and dried (desiccator, 24 h). For the base layer, PVP K90 (2.45 g) and carboxymethyl chitosan (50 mg) were dissolved in deionized water (10 mL), added into the mould, and centrifuged (3000 rpm, 25 ℃, 4 min). After drying (24 h), microneedles (PPLG@MN) were extracted. Similarly, LG@MN (L-arg/GOx-loaded), PPB@MN (PPB-loaded), and Blank@MN (no drug) were prepared.

### Chemical properties

The particle size and zeta potential of PPB and PPLG were verified using Malvern particle size analyzer (Malvern Instruments Ltd, UK). The morphology and structure of PPB and PPLG were characterized by transmission electron microscopy (TEM; Hitachi H7700), as well as the surface morphology of the MN was investigated using scanning electron microscopy (SEM; Thermo Scientific Apreo-2C). Then, UV spectroscopy and Fourier transform infrared spectroscopy were used to determine the UV and IR profiles of the nanozymes to characterize whether they were successfully synthesized. X-ray powder diffraction (XRD) data were acquired on a Bruker D8 ADVANCE in the angular range corresponding to 2θ = 20-80^◦^. Together with an X-ray photoelectron spectrometer (XPS, ESCALAB MK II), the elemental valence and chemical composition of PPLG were characterized. The distribution of elements in PPLG was characterized using energy dispersive spectroscopy (EDS, Oxford Max-65) mapping analysis. PPLG was dispersed in phosphate-buffered saline (PBS, pH 7.4) and 10% fetal bovine serum (FBS) at a final concentration of 100 μg/mL. The samples were incubated at 37 °C, and aliquots were collected at various time points from 0 to 72 h. Particle size was measured using a particle size analyzer, while transmittance was recorded using a UV-Vis spectrophotometer to assess the stability of PPLG.

### Characterization of PPLG@MN

The mechanical properties of PPLG@MN and Blank@MN were assessed using a texture analyzer (CT3, Brookfield, USA). Samples were placed tip-up on the stage with a trigger force of 0.05 N, loading force of 50 N, and compression rate of 0.5 mm/s. Needle height was measured *via* microscope. For penetration studies, PPLG@MN was prepared with 0.4% Trypan blue (replacing water) as the tip solvent. Depilated skin was placed stratum corneum-up, pressed with microneedles for 30 s, and removed after 3 min to assess pore formation. To evaluate the storage stability of GOx in the microneedle system, PPLG@MN patches were stored at 4 °C (or 25 °C). Samples were collected on days 0, 7, 14, 21, and 28. At each time point, the microneedles were dissolved in PBS, and GOx activity was quantified using a commercial GOx activity assay kit (Beyotime Biotechnology, China; P2747S) by measuring hydrogen peroxide generation. The residual activity was calculated as a percentage of the initial activity on day 0.

### *In vivo* dissolution study of PPLG@MN

After removing the hair on the back of the mice, PPLG@MN was pressed onto the skin for different durations (0, 0.5, 1, 2, and 3 min, respectively). The samples were removed at each time point and the remaining length of the needle was measured using a microscope. Based on the data obtained, a dissolution trend line was plotted with time as the horizontal axis and percentage of remaining needle length as the vertical axis.

### Validation of enzyme-mimetic and cascade catalytic activities

The catalase-like activity of Prussian blue nanozymes was evaluated under simulated physiological conditions by measuring dissolved oxygen levels in glucose solution at various time points H_2_O_2_ production was quantified using a hydrogen peroxide detection kit across varying glucose concentrations. Similarly, NO release was measured with an NO detection kit, where PPLG was reacted with different glucose concentrations, and levels were determined using NO standard curves at specified time intervals. These results confirm the efficient progression of the cascade reaction.

### *In vitro* anti-inflammatory effect

RAW 264.7 cells were incubated with LPS for 24 h and then co-cultured with each treatment group for another 24 h. Afterwards, the cells were co-incubated with FITC fluorescent dye under dark conditions for 30 min and visualized with a fluorescence microscope. The generated ROS were quantified by flow cytometry. RAW 264.7 cells were pretreated with LPS for 24 h. Then, different samples were added for further incubation for 24 h. Supernatants were collected. The anti-inflammatory effect of PPLG was further investigated using an enzyme-linked immunosorbent assay kit.

### *In vitro* antibacterial activity

*S. aureus* (ATCC 6538) and *P. aeruginosa* (ATCC 9027) were purchased from Guangdong Microbial Strain Collection Centre. These bacteria were cultured in LB broth at 37 ℃, and then the concentration of each bacterial suspension was adjusted to 5 × 10^6^ CFU/mL. Live-dead staining was used to assess the viability of *S. aureus* and *P. Aeruginosa* after antimicrobial activity. Bacteria were incubated with different samples at 37 °C for 24 h, followed by live-dead bacterial staining. In short, 1 mL of bacterial suspension was incubated with SYTO-9 and PI staining in the dark at 37 ℃ for 30 min. The stained bacterial solution was then dropped on a slide and fluorescent images were taken using an inverted fluorescence microscope.

### Assessment of biofilm disrupting activity

The anti-biofilm efficacy of different treatment groups was evaluated using live/dead staining (SYTO-9/PI) and crystal violet staining. Then, biofilm formation: Bacterial cultures in the logarithmic growth phase were incubated at 37 °C for 48 h to establish adherent biofilms. Firstly, different samples were added to the biofilm and the mixed solution was incubated at 37 °C for 24 h. After incubation the supernatant was removed and the bottom biofilm was washed three times with PBS then the biofilm was treated by crystal violet staining and quantitatively analyzed by measuring the absorbance at 590 nm using a microplate reader. After incubation with SYTO-9 and PI (5 μg/mL) staining for 30 min, and photographed by CLSM to obtain three-dimensional fluorescence.

### *In vitro* biocompatibility

The cytocompatibility of MN was tested by MTT assay using HUVEC and RAW 264.7 cells. Cells (5 × 10^3^/well) were placed in 96-well plates and cultured in DMEM with 10% FBS at 37 °C under 5% CO_2_ for 24 h. After treatment with test compounds for 24 h, cells were washed with PBS, incubated with 20 μL MTT (4 h), and absorbance was measured at 450 nm using a microplate reader. Co-incubation of HUVEC and RAW 264.7 with 100 μM H_2_O_2_ for 24 h was used as a model group. Cultured cells were stained using the Live and Dead Survival/ Cytotoxicity Assay Kit for 30 min at 37 °C. Finally, cells were observed using an inverted fluorescence microscope.

### Assessment of endothelial cell migration

To verify the effect of PPLG@MN on endothelial cell migration, HUVEC migration was assessed using a scratch assay. The cell scratch experiment first adjusted the cell density of HUVEC to 2 × 10^6^ cells/mL and inoculated them in 6-well culture plates. After monolayer formation, uniform scratches were created using sterile pipette tips. Following PBS washes to remove debris, treatments were applied. Scratch closure was monitored at 0, 24, and 48 h using phase-contrast microscopy. Migration rates were quantified by measuring scratch area reduction (%) using ImageJ, calculated as:

Migration rate (%) = (initial scratch area - scratch area) / initial scratch area × 100%. HUVEC (1×10^5^ cells/mL) were seeded in the upper chamber (200 μL/well, n = 3), with 600 μL complete medium in the lower chamber. After 24 h incubation, non-migrated cells were removed by PBS washing. Cells were fixed with 4% paraformaldehyde (30 min), stained with crystal violet, and imaged under an inverted microscope. Migrated cells in random fields were counted using ImageJ to quantify migration rates.

### *In vitro* tube formation assay

Add 200 μL of Matrigel matrix to each well of a 24-well plate. After adding the matrix gel, the plate was placed back into the cell culture incubator and let it stand for 30 min. HUVECs were digested to prepare a cell suspension at 1 × 10^6^/mL. A total of 100 μL of cell suspension was added to each well, and materials were co-cultured with the cells at the same time, followed by staining after 4 h. Tube formation images were obtained using CLSM. Total length and node quantification were analyzed by Image J.

### Establishment of diabetic mouse model

Male C57BL/6J mice (6-8 weeks old) were acclimated for one week prior to experimentation. Diabetes was induced by intraperitoneal injection of streptozotocin (STZ, 50 mg/kg in 1% citrate buffer) once daily for five consecutive days. After a three-day interval, blood glucose levels were measured from tail vein samples twice per week. Mice with fasting blood glucose levels exceeding 11.7 mmol/L on three consecutive measurements were considered diabetic and included in subsequent experiments.

### *In vivo* wound healing experiments

Mice with successful model construction were randomly divided into eight groups, (NC, Model, PPB, PPB@MN, LG, LG@MN, PPLG, PPLG@MN). Under anesthesia, an 8-mm full-thickness wound was created with 20 μL bacteria suspension (1×10^6^ CFU/mL), and covered with sterile dressing. Treatment began upon formation of a yellowish mucus layer (saline for NC/Model groups). Wound areas were photographed on days 0, 3, 7, and 14 for closure rate analysis (ImageJ). Bacterial loads were quantified via plate counting of traumatized tissue on day 7. Mice in the experimental group were administered the respective therapeutic agents on Day 0 after biofilm formation (one microneedle patch per wound, or an equivalent volume of therapeutic drug solution). Mice in the NC group and Model group were treated with 3M wound dressings of the same size.

Wound closure rate (%) = (S₀ - S) / S₀ × 100%, where S₀ is the wound area on day 0 and S is the wound area at the observation time point.

### Histopathological and inflammatory tests

Three mice were randomly selected and euthanized on the 7th day after drug administration, with their dorsal tissues collected for histological analysis. All mice were sacrificed by cervical dislocation on the 14th day after drug administration, and their dorsal skin and visceral organs were harvested for histological analysis and safety evaluation. Skin regeneration at the wound site was evaluated by histomorphological analysis and assessment of inflammatory markers. Skin tissues were fixed in 4% paraformaldehyde and subjected to hematoxylin and eosin (H&E), Masson' s trichrome, immunofluorescence, and immunohistochemical staining. H&E staining was used to assess epidermal thickness, hair follicle density, and epithelial regeneration. Collagen deposition and wound closure were evaluated by Masson' s trichrome staining. To investigate neoangiogenesis and vascular maturation, double immunofluorescence staining was performed using endothelial marker CD31 and pericyte marker α-SMA. Macrophages were initially localized using the macrophage-specific marker (F4/80), and further identified based on the M1-type marker (iNOS) and M2-type marker (CD206). Macrophage polarization and resolution of prolonged inflammation were further analyzed by triple immunofluorescence staining targeting macrophage phenotypes.

### Statistical analysis

All data are presented as the mean ± standard deviation (SD) from at least three independent parallel experiments. Statistical analysis of the data was performed using unpaired t-test and one-way analysis of variance (ANOVA). All statistical analyses were performed using GraphPad Prism 9.0 or Origin software. 'ns' indicates non-significant (*p* > 0.05), '^*^' indicates *p* < 0.05, '^**^' indicates *p* < 0.01, '^***^' indicates *p* < 0.001 *vs.* control group. ^'&'^ indicates *p* < 0.05,^ '&&'^ indicates *p* < 0.01, ^'&&&'^ indicates *p* < 0.001 for indicated comparisons.

## Results and Discussion

### Fabrication and characterization of the PPLG

**Figure 1:**
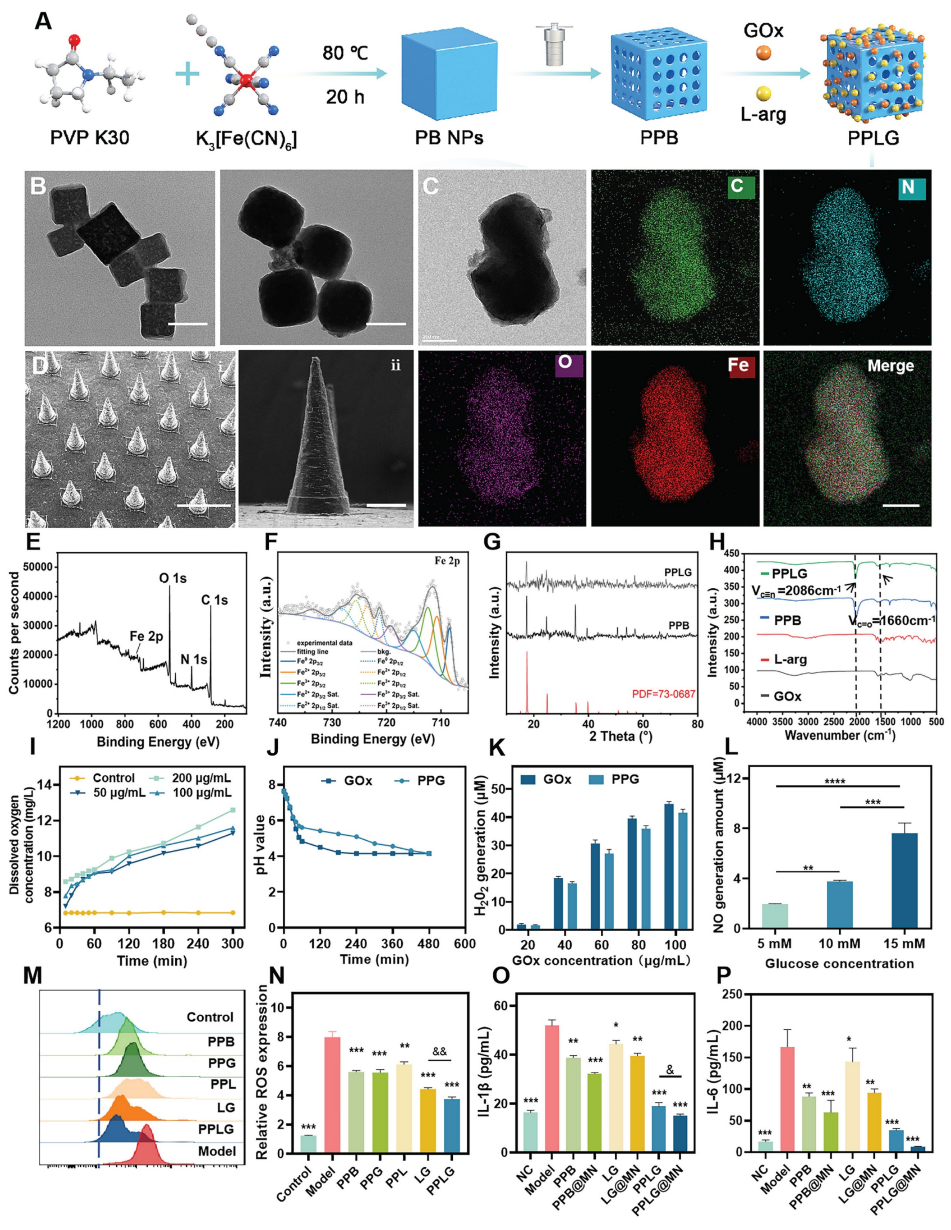
Characterization of PPLG nanozymes. (A) Synthesis scheme of the PPLG nanozymes. (B) TEM images of PPB and PPLG (scale bar: 100 nm). (C) EDS mappings in selected areas of PPLG (scale bar: 200 nm). (D) SEM images: (i) side view (scale bar: 1 mm); (ii) a single PPLG@MN (scale bar: 200 μm). (E) XPS spectrum of PPLG. (F) High-resolution Fe 2p XPS spectrum. (G) Comparative XRD patterns of PPLG, PPB and standard PB. (H) FTIR spectra of PPLG, PPB, GOx and L-arg. (I) Catalytic O_2_ generation by PPB at varying concentrations in H_2_O_2_ solution (n = 3). (J) Temporal pH profile under different conditions (n = 3). (K) Glucose concentration-dependent H_2_O_2_ production by GOx and PPG (n = 3). (L) Glucose-mediated NO generation by PPLG (n = 3). (M) Flow cytometry results for ROS quantification. (N) Quantification of flow cytometry data (n = 3). (O, P) IL-1β and IL-6 levels by ELISA (n = 6). Values are expressed as the mean ± SD. '^*^' indicates *p* < 0.05, '^**^' indicates *p* < 0.01, '^***^' indicates *p* < 0.001. '^&^' indicates *p* < 0.05, '^&&^' indicates *p* < 0.01, '^&&&^' indicates *p* < 0.001.

In this study, PB and PPB nanoparticles were synthesized *via* hydrothermal and chemical etching methods. Subsequently, L-arg and GOx were successfully loaded onto PPB, as illustrated in Figure 1A. Quantitative results showed that the adsorption rate of GOx was 79.82% ± 0.62%, and the loading capacity of L-Arg was 12.5% ± 0.3%. The synthesized PPB exhibited a uniform size distribution and orthorhombic morphology. Upon GOx encapsulation, the morphology of the resulting PPLG nanoparticles transitioned from cubic to smooth spheroidal structures (Figure 1B). Elemental mapping analysis (Figure 1C) indicated a homogeneous distribution of Fe, C, O, and N elements, confirming successful GOx and L-arg loading onto PPB. X-ray photoelectron spectroscopy (XPS) analysis further verified the chemical composition of the nanozymes, detecting clear peaks corresponding to C, O, N, and Fe elements in the PPLG structure. High-resolution XPS analysis (Figure 1E-F) exhibited characteristic peaks at 712.31 eV (Fe^3+^ 2p_3/2_) and 723.48 eV (Fe^2+^ 2p_1/2_), respectively. Collectively, these results confirm the successful preparation of PPLG. As shown in Figure S1, particle size increased from 100 nm to 140 nm with a concomitant decrease in zeta potential from 17.2 mV to 7.4 mV post-drug loading, which may be attributed to drug loading and modification. PPLG maintained stable particle size and optical properties in both 10% FBS and PBS, indicating its satisfactory physicochemical stability under physiologically relevant conditions (Figure S2). XRD analysis (Figure 1G) demonstrated that PPB maintained characteristic diffraction peaks of standard Prussian blue nanoparticles, albeit with reduced intensity, indicating diminished crystallinity but preservation of the crystalline framework post acid-etching modification. FTIR analysis (Figure 1H) confirmed characteristic absorption bands at 2086 cm^-1^ (C≡N stretching) and 1660 cm^-1^ (C=O stretching) in both PPB and PPLG, confirming the structural integrity of the Prussian blue framework. UV-Vis absorption spectra (Figure S3) exhibited characteristic near-infrared absorption at 700 nm, corresponding to the Fe Ⅱ-C≡N-Fe Ⅲ electron transition. Notably, PPLG exhibited reduced UV absorption intensity and a blue-shifted maximum absorption peak compared to PPB, indicating the successful incorporation of GOx and L-arg. Collectively, these results demonstrate the successful synthesis of a GOx/L-arg-loaded PPB nanosystem. Based on previous studies 39,40, PPLG@MN was fabricated using a two-step micromolding process (Figure S4). As shown in Figure S5-S6, the PPLG@MN exhibited a smooth conical shape, with a blue-tipped appearance. The microneedles demonstrated appropriate dimensional parameters and enhanced surface roughness post-PPLG loading, facilitating effective biofilm penetration (Figure 1D). Mechanical integrity analysis (Figure S7-S8) revealed minimal height reduction for both blank MN (16.8% ± 0.3%) and PPLG@MN (17.2% ± 0.4%) (*p* > 0.05), indicating preserved mechanical properties after drug loading. Skin insertion capability assessed *via* trypan blue staining confirmed pore formation efficiency exceeding 95% (Figure S9). Histological evaluation further verified a consistent penetration depth of approximately 400 ± 25 μm (Figure S10), supporting the effective transdermal delivery capability of PPLG@MN. Dissolution kinetics analysis (Figure S11) revealed complete microneedle degradation within 3 min post-insertion, enabling rapid drug release through the viscous biofilm barrier characteristic of diabetic wounds. In addition, long-term storage stability tests of GOx (Figure S12) showed that PPLG@MN retained high enzymatic activity after 28 days of storage at both 4 °C and 25 °C, further confirming that the microneedle system effectively preserves the biological activity of the enzyme. Collectively, these results demonstrate that PPLG@MN possesses excellent mechanical strength, rapid dissolution performance, efficient transdermal delivery capability, and favorable enzyme stability, thereby supporting its potential for treating infected wounds.

### Performance validation of cascade nanozymes

Prussian blue nanozymes have garnered significant attention in recent years due to their superior enzyme-like activity and anti-inflammatory properties 41. To verify the catalase-like activity of PPB, dissolved oxygen quantification assays were performed PPB demonstrated concentration-dependent oxygen generation (Figure 1I), confirming its capacity to continuously supply oxygen for cascade reactions. Intracellular ROS analysis revealed that PPB effectively normalized elevated ROS levels in LPS-stimulated cells to near-control values (*p* > 0.05 *vs.* control) (Figure S13). Enzyme activity preservation is crucial for cascade reaction efficiency. pH monitoring confirmed that GOx-incorporated PPB maintained comparable enzymatic activity to free GOx (Figure 1J), indicating preserved biological function post-encapsulation. Glucose concentration-dependent H_2_O_2_ production by PPG (PPB + GOx) (Figure 1K) confirmed sufficient substrate availability for subsequent catalytic steps.

The glucose-responsive NO generation profile of PPLG (Figure 1L) demonstrated successful cascade reaction progression, with statistically significant (*p* < 0.01) concentration-dependent NO production, confirming system sensitivity to glucose concentration variations. Fluorescence imaging showed markedly elevated intracellular green fluorescence intensity in LPS-stimulated cells (Figure S14), which significantly decreased upon treatment, with the most pronounced reduction observed in the PPLG-treated group. Flow cytometry confirmed these observations (Figure 1M-N), highlighting enhanced antioxidant and anti-inflammatory effects of PPLG, surpassing those of PPB alone. ELISA of RAW264.7 cell supernatants indicated superior anti-inflammatory efficacy of PPLG relative to PPB and the L-arg/GOx (LG) groups under hyperglycemic conditions (Figure 1O-P). Notably, the LG group showed moderate anti-inflammatory effects, though significantly less pronounced than other formulations (*p* < 0.05). Importantly, DMN incorporation significantly enhanced the anti-inflammatory potential across all treatment groups. Biocompatibility assays of microneedle excipients (Figure S15) and *in vivo* puncture safety tests (Figure S16) confirmed the excellent biosafety and minimal skin irritation potential of the PPLG@MN. Skin recovery occurred rapidly post-application, with no evident trauma after one hour.

### *In vitro* evaluation of antibacterial activity

**Figure 2:**
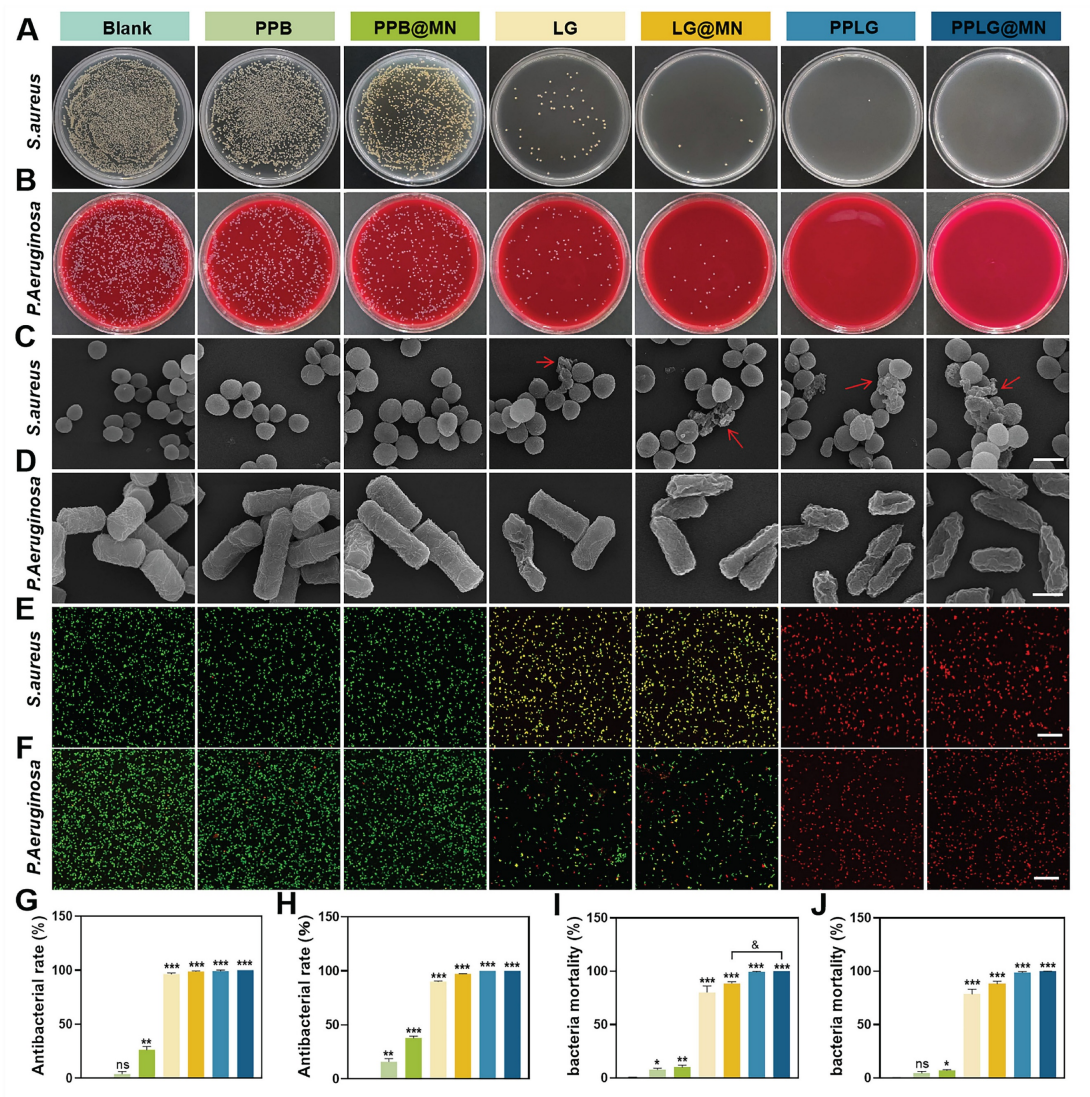
*In vitro* antibacterial activity of cascade nanoenzymes. (A, B) Photographs of *S. aureus* and *P. aeruginosa* colonies on LB agar plates and blood plates. (C, D) Representative SEM images of *S. aureus* and *P. aeruginosa* after various treatments (scale bar: 1 μm). (E, F) Live/dead fluorescence staining images of *S. aureus* and *P. aeruginosa* after treatment (Green, live bacteria; red, dead bacteria; scale bar: 20 μm). (G, H) Inhibition rates of *S. aureus* and *P. aeruginosa* after different treatments (n = 3). (I, J) Bacterial mortality of *S. aureus* and *P. aeruginosa* stained images by live/dead fluorescence (n = 3). Values are expressed as the mean ± SD. '^*^' indicates *p* < 0.05, '^**^' indicates *p* < 0.01, '^***^' indicates *p* < 0.001; '^&^' indicates *p* < 0.05, '^&&^' indicates *p* < 0.01, '^&&&^' indicates *p* < 0.001.

Bacterial infections commonly occur at the initial stages of skin injury, prolonging inflammation and contributing to chronic wound formation. To assess antibacterial efficacy, *Staphylococcus aureus* (*S. aureus*, Gram-positive) and *Pseudomonas aeruginosa* (*P. aeruginosa*, Gram-negative) were selected as model pathogens. The antibacterial activity of each formulation was evaluated *via* plate coating assays. The PPB group exhibited minimal bacteriostatic effects (~5% inhibition). In contrast, formulations incorporating LG demonstrated substantial inhibition due to NO production (Figure 2A-B). SEM revealed intact bacterial morphology in the PPB and PPB@MN groups, similar to the untreated controls (Figure 2C-D). In contrast, considerable morphological damage, including cell rupture and content leakage, was evident in LG, LG@MN, PPLG, and PPLG@MN-treated groups, particularly pronounced with PPLG formulations, signifying robust, broad-spectrum antibacterial efficacy. Live/dead bacterial assays further confirmed these observations (Figure 2E-F). Notably, PPLG and PPLG@MN groups achieved nearly complete bacterial eradication (~100%; Figure 2G-H), with quantitative analysis consistently ranking antibacterial efficacy as PPLG@MN ≈ PPLG ≥ LG@MN ≈ LG > PPB@MN > PPB (Figure 2I-J).

### Biofilm destruction efficiency

**Figure 3:**
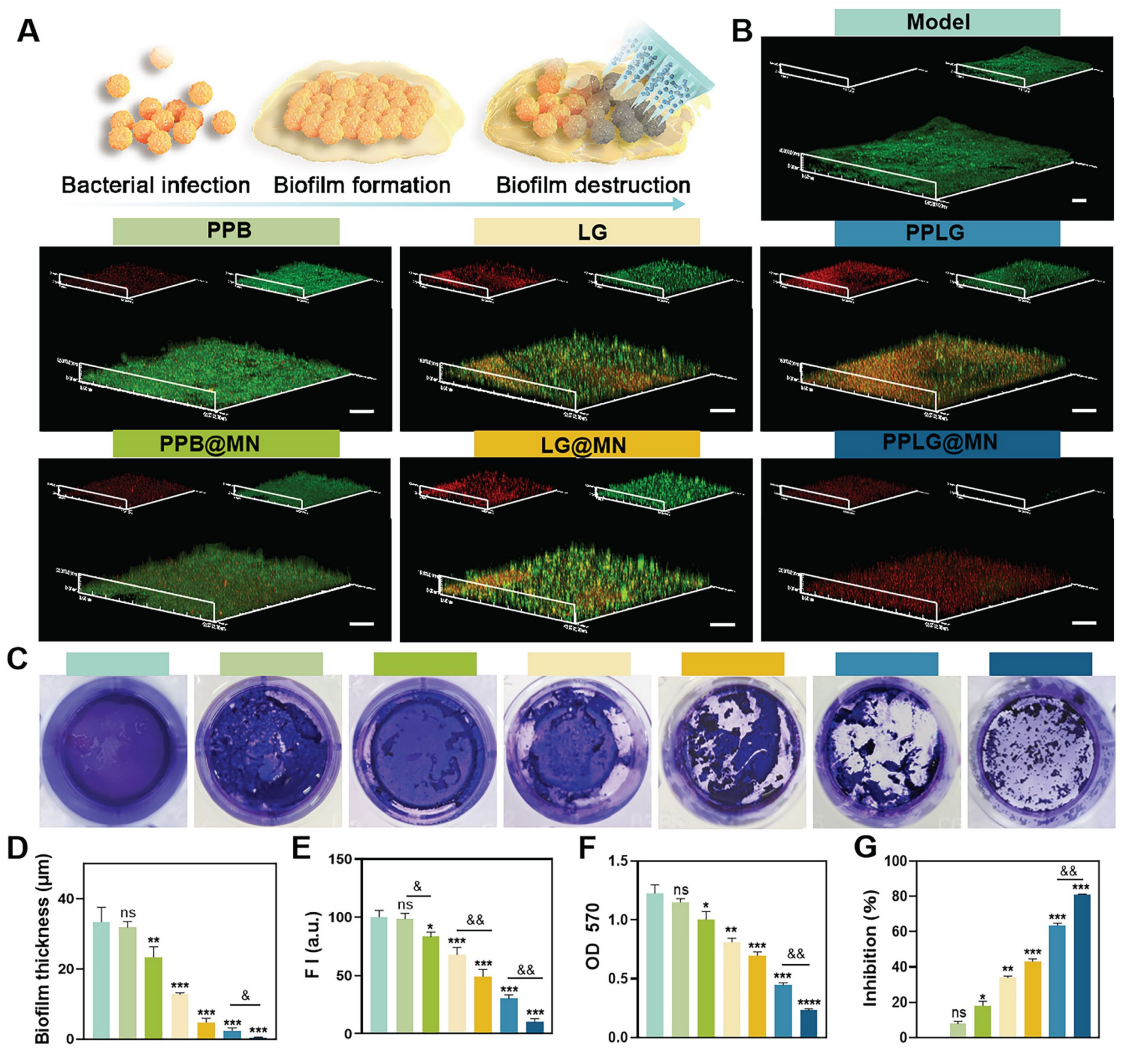
Cascading nanoenzymes disrupt biofilms effectively. (A) Schematic illustration of the PPLG@MN mechanism against biofilms. (B) Live/dead staining of *S. aureus* biofilms in different treatment groups (scale bar: 20 μm; n = 3). (C) Crystal violet staining of mature biofilms (n = 6). (D) Quantification of biofilm thickness across groups (n = 3). (E) Quantification of biofilm metabolic activity based on fluorescence intensity (n = 3). (F, G) OD_570_ measurements and inhibition rates of biofilms in each group (n = 6). Values are expressed as the mean ± SD. '^*^' indicates *p* < 0.05, '^**^' indicates *p* < 0.01, '^***^' indicates *p* < 0.001; '^&^' indicates *p* < 0.05, '^&&^' indicates *p* < 0.01, '^&&&^' indicates *p* < 0.001.

Bacterial biofilms (BF) significantly impede wound healing, forming dense extracellular polymeric substance (EPS) matrices that resist immune and antibiotic penetration, greatly diminishing therapeutic effectiveness. Microneedles, by virtue of their unique structure, are characterized by physical penetration and efficient delivery, disrupting the biofilm. The proposed mechanism of MN-mediated biofilm disruption is illustrated in Figure 3A. Confocal microscopy images indicated robust, intact biofilms predominantly composed of viable bacteria in control, PPB, and PPB@MN groups (Figure 3B), whereas LG formulations exhibited moderate anti-biofilm effects attributed to NO-mediated bactericidal activity. Crystal violet staining further validated the biofilm-destroying ability of PPLG@MN (Figure 3C), with a substantial reduction in purple coloration observed in LG, LG@MN, PPLG, and PPLG@MN groups. Quantitative analysis confirmed that compared with conventional dressings, both biofilm thickness (Figure 3D) and green fluorescence intensity (Figure 3E) were significantly reduced in the MN-treated group (*p* < 0.01), demonstrating enhanced biofilm disruption and prevention of bacterial aggregation. Spectrophotometric quantification revealed biofilm destruction rates of 34.4% ± 2.1%, 43.5% ± 1.8%, 63.8% ± 2.3%, and 81.1% ± 1.9% for LG, LG@MN, PPLG, and PPLG@MN groups, respectively (Figure 3F-G), confirming the combined physical disruption mediated by MN and superior biofilm eradication by PPLG@MN.

### *In vitro* biocompatibility evaluation of cascade nanozymes

**Figure 4:**
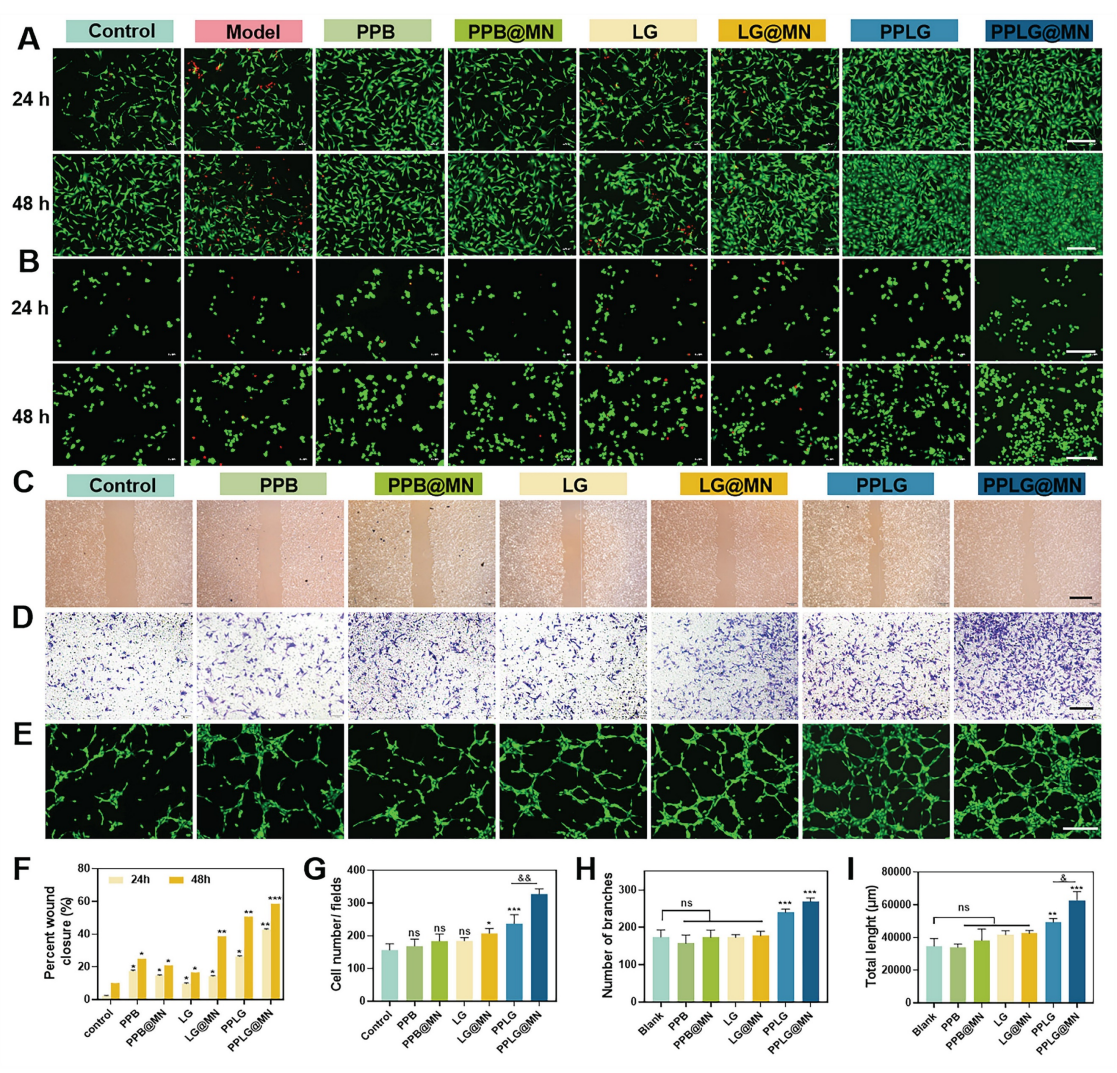
Cellular safety, pro-migration, and pro-angiogenic effects of cascade nanozymes. (A, B) Live/dead staining of HUVEC and RAW 264.7 cells after 24 and 48 h of incubation with various samples (scale bar: 200 μm). (C) Cell migration of HUVEC in different groups at 48 h (scale bar: 2 mm). (D) Transwell staining images of HUVEC after 24 h incubation in each drug treatment (scale bar: 2 mm). (E) Tube formation capacity of HUVEC after 6 h incubation with different preparations (scale bar: 200 μm). (F) Cell scratch healing rate (n = 3); (G) Transwell migration counts of HUVEC after 12 h (n = 3). (H, I) Nodal branching and total length results for tube formation capacity (n = 3). Values are expressed as the mean ± SD. '^*^' indicates *p* < 0.05, '^**^' indicates *p* < 0.01, '^***^' indicates *p* < 0.001; '^&^' indicates *p* < 0.05, '^&&^' indicates *p* < 0.01.

Biocompatibility is essential for the clinical translation of biomaterials. In this study, we systematically evaluated the biocompatibility of the cascade nanozymes through MTT assays and live/dead staining using HUVECs and RAW264.7 cells. As shown in Figure S17, both PPB and PPLG exhibited extremely low cytotoxicity at concentrations up to 200 μg/mL. Live/dead staining further confirmed that cells in the PPLG@MN group maintained normal morphology and proliferative capacity (Figure 4A-B). In contrast, both the model group and the LG group exhibited marked cell death, with the cytotoxicity in the LG group nearly identical to that of the model group (Figure S18). Together with the evidence in Figure 1K showing that GOx retains strong catalytic activity within the formulation and continuously generates H_2_O_2_, it can be concluded that the cellular damage observed in the LG group is primarily caused by the acute oxidative stress induced by the burst-like accumulation of H_2_O_2_ during glucose oxidation. Conversely, the cascade reaction design within the PPLG@MN system enables effective modulation of both O_2_ and NO generation, thereby preventing excessive H_2_O_2_ buildup, maintaining intracellular homeostasis, and promoting cell survival.

### Pro-migratory and angiogenic capacity of cascade nanozymes

Enhanced endothelial migration and angiogenesis are critical for effective wound healing. Scratch wound assays demonstrated that PPLG@MN significantly alleviated hyperglycemia-induced impairment of HUVEC migration compared to other groups (Figure 4C, F). Transwell experiments confirmed enhanced endothelial cell migration under hyperglycemic conditions in the PPLG@MN group (Figure 4D, G), with quantitative analysis indicating improved migratory ability following PPLG loading onto MN. These findings suggest that PPLG@MN promotes both horizontal and vertical HUVEC migration. Given the essential role of rapid revascularization in tissue repair, we evaluated angiogenic potential through tube formation assays. Both PPLG and PPLG@MN groups showed extensive tubular network formation, significantly exceeding vascular density in the control group (Figure 4E). In contrast, PPB formulations showed no angiogenic effect, and LG groups failed to achieve substantial angiogenesis despite NO generation. Quantitative analysis of branch number and tube length confirmed the superior angiogenic capacity of PPLG formulations (Figure 4H-I), attributable to glucose-responsive NO release and enhanced angiogenic activity of the cascade nanozyme system.

### *In vivo* treatment of diabetic biofilm wounds

**Figure 5:**
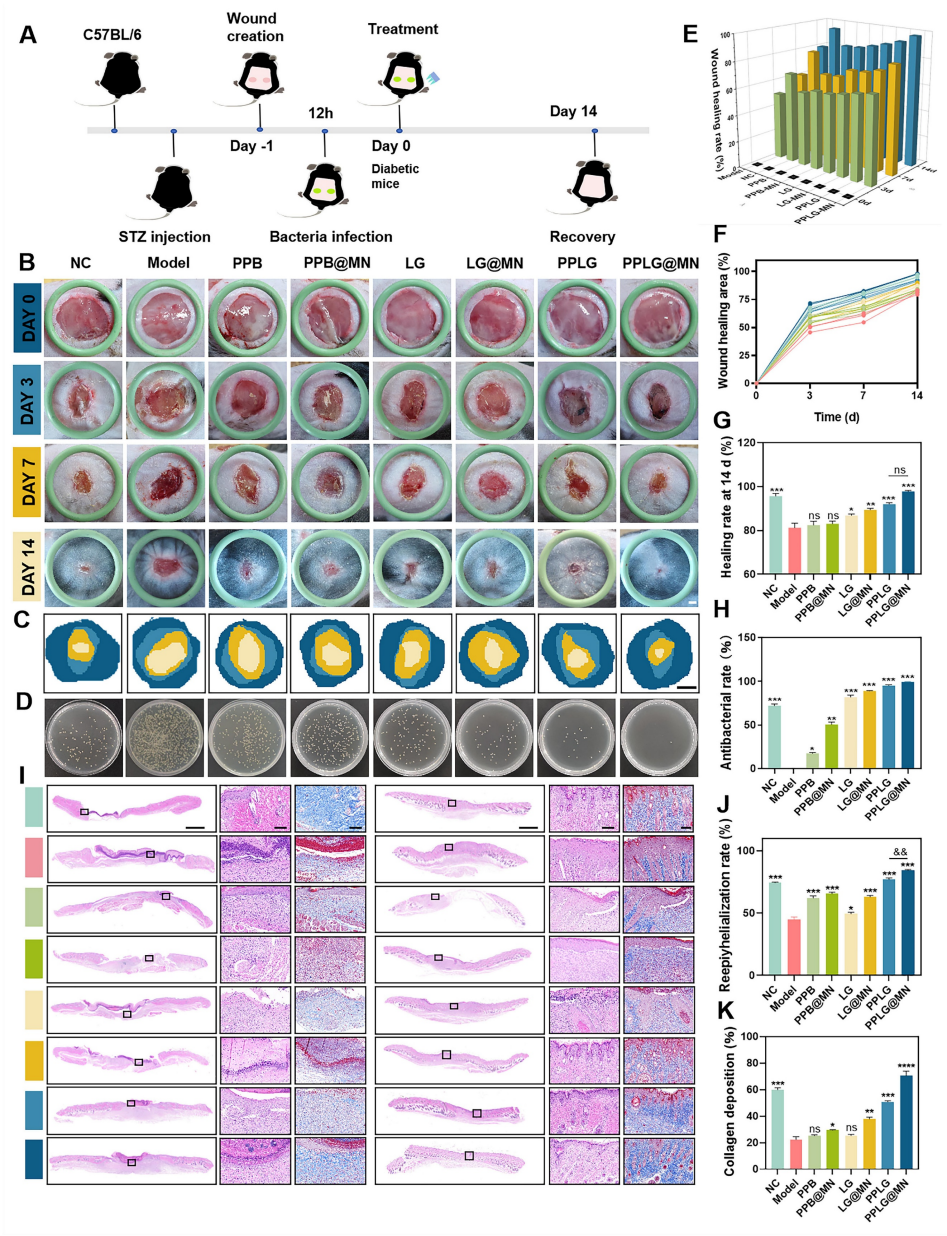
Effect of wound healing in diabetic biofilm mice *in vivo*. (A) Schematic diagram of experimental wound healing in biofilm diabetic mice treated with PPLG@MN. (B) Photographs of the wounds of the C57BL/6 mice after various treatments at different time points (scale bar: 1 mm). (C) Dynamic healing of the wound area at different times (scale bar: 2 mm). (D) Photographs of the corresponding* S. aureus* colonies on LB agar plates in wounds on day 3 after treatment. (E) Wound shrinkage quantified over a 14-day treatment period (n = 6). (F) Changes in wound healing rates (n = 6). (G) The wound healing rate was calculated at day 14 post-treatment (n = 6). (H) Antimicrobial efficacy within the wound site was evaluated on day 3 using standardized microbiological assays (n = 3). (I) Representative H&E staining images of day 7, 14 wounds (scale bar: 1 mm for panoramic view, 100 μm for magnified view). (J) The epithelial regeneration rate (%) quantified to evaluate the extent of re-epithelialization in each treatment group (n = 3). (K) The collagen deposition rate (%) measured to assess extracellular matrix remodeling (n = 3). Values are expressed as the mean ± SD. '^*^' indicates *p* < 0.05, '^**^' indicates *p* < 0.01, '^***^' indicates *p* < 0.001; '^&^' indicates *p* < 0.05, '^&&^' indicates *p* < 0.01.

Chronic diabetic wounds typically exhibit persistent biofilms, inflammation, and impaired healing due to hyperglycemia-induced cellular dysfunction, angiogenic inhibition, and pro-inflammatory cytokine secretion. These factors collectively impede effective therapeutic intervention and prolong wound healing. We designed an* in vivo* diabetic biofilm-infected wound model (Figure 5A), randomizing mice into eight groups: (1) NC (non-diabetic control), (2) Model (diabetic infection model), (3) PPB, (4) PPB@MN, (5) LG, (6) LG@MN, (7) PPLG, and (8) PPLG@MN. Wound progression was documented at predetermined intervals (0, 3, 7, and 14 days post-surgery), with wound area quantification performed using ImageJ software. Persistent hyperglycemia was maintained across all treatment groups throughout the study period (Figure S19). Notably, PPLG@MN-treated mice exhibited significant weight recovery compared to the model group, indicating improved systemic conditions (Figure S20). Wound healing analysis revealed that PPLG@MN treatment achieved comparable or superior healing outcomes relative to the non-diabetic NC group, with >95% wound closure by day 14 and high levels of re-epithelialization and no observable hypertrophic features (Figure 5B). The superior efficacy of PPLG@MN is likely due to its multifunctional therapeutic capabilities: inherent anti-inflammatory effects from PPB, effective antimicrobial activity mediated by the glucose-responsive NO cascade, and enhanced biofilm penetration with targeted delivery facilitated by MN.

The wound healing process was comprehensively evaluated following the sequence of Figure 5C-G. First, schematic representations of wound repair (Figure 5C) and bacterial burden assessment (Figure 5D) illustrated the overall therapeutic procedure and microbiological outcomes. Additional schematic diagrams (Figure 5E) further displayed the dynamic wound-healing progression across different treatment groups. Subsequently, quantitative measurements of wound area at 0, 3, 7, and 14 days (Figure 5F) revealed that the PPLG@MN group achieved comparable or superior wound closure (97.1% ± 1.2%) relative to the NC group (95.3% ± 1.5%), and markedly outperformed the model group (77.3% ± 2.1%). The calculated healing rates (Figure 5G) confirmed that PPLG@MN effectively reversed hyperglycemia-induced impairment and restored normal tissue repair. Meanwhile, bacterial culturing of wound tissues on day 3 (Figure 5D) demonstrated the enhanced antimicrobial activity of PPLG formulations, with PPLG@MN achieving ~99% clearance. The LG cascade treatment also exhibited noticeable antibacterial effects. Similar to the *in vitro* experiments, these results indicated that the NO released from the cascade reaction can effectively eliminate bacteria and accelerate wound healing, and that MN can break through the biofilm barrier to achieve targeted drug delivery to the lesion for therapeutic effect.

*In vitro* hemolysis assays indicated clear supernatants for all formulations except LG and LG@MN, which showed mild hemolysis (Figures S21-S22). Quantitative hemolysis assessment confirmed hemolysis rates below 5% for PPB and PPLG groups, aligning with international biomaterial safety standards. The observed hemolysis with LG formulations likely resulted from rapid NO burst release, causing indiscriminate cellular damage. In contrast, sustained NO release from PPLG-based formulations maintained cellular integrity, indicating excellent biocompatibility and translational potential for chronic diabetic wound treatment.

### Histopathological analysis of diabetic biofilm wounds

Histopathological evaluation of diabetic wound healing was conducted using H&E and Masson's trichrome staining. Quantitative analysis revealed significant differences in wound healing progression among treatment groups. By day 7 post-treatment, the PPLG@MN group demonstrated superior wound healing characteristics, including: (1) extensive neovascularization within granulation tissue, (2) partial epithelialization, and (3) reduced inflammatory infiltration compared to other treatment groups (Figure 5I). Notably, the PPLG@MN group exhibited enhanced wound closure parameters exceeding those observed in the NC group (*p* < 0.05). By day 14, histological examination showed that the PPLG@MN group exhibited substantially restored epidermal structure, with tissue architecture closely resembling adjacent normal skin. In contrast, the other groups displayed characteristic pathological features of impaired wound healing, including epidermal hyperplasia, hyperkeratosis, and persistent inflammatory infiltration. Quantitative assessment of re-epithelialization and follicular regeneration demonstrated significantly higher values in the PPLG@MN group compared to controls (Figure 5J, Figure S23; *p* < 0.01) Collagen deposition analysis revealed temporal and spatial differences in extracellular matrix remodeling (Figure 5K). By day 7, only the PPLG@MN group exhibited organized collagen fiber deposition, while other groups showed either absent or disorganized matrix formation. At day 14, the NC, PPLG and PPLG@MN groups had more complete collagen deposition with dense blue-stained collagen fibers. Among them, the PPLG@MN group not only had dense blue-stained collagen deposition, but also significantly higher numbers of hair follicles, indicating that the PPLG@MN group exhibited the best collagen remodeling and the most effective promotion of wound healing.

### PPLG@MN promotes wound healing mechanisms* in vivo*

**Figure 6:**
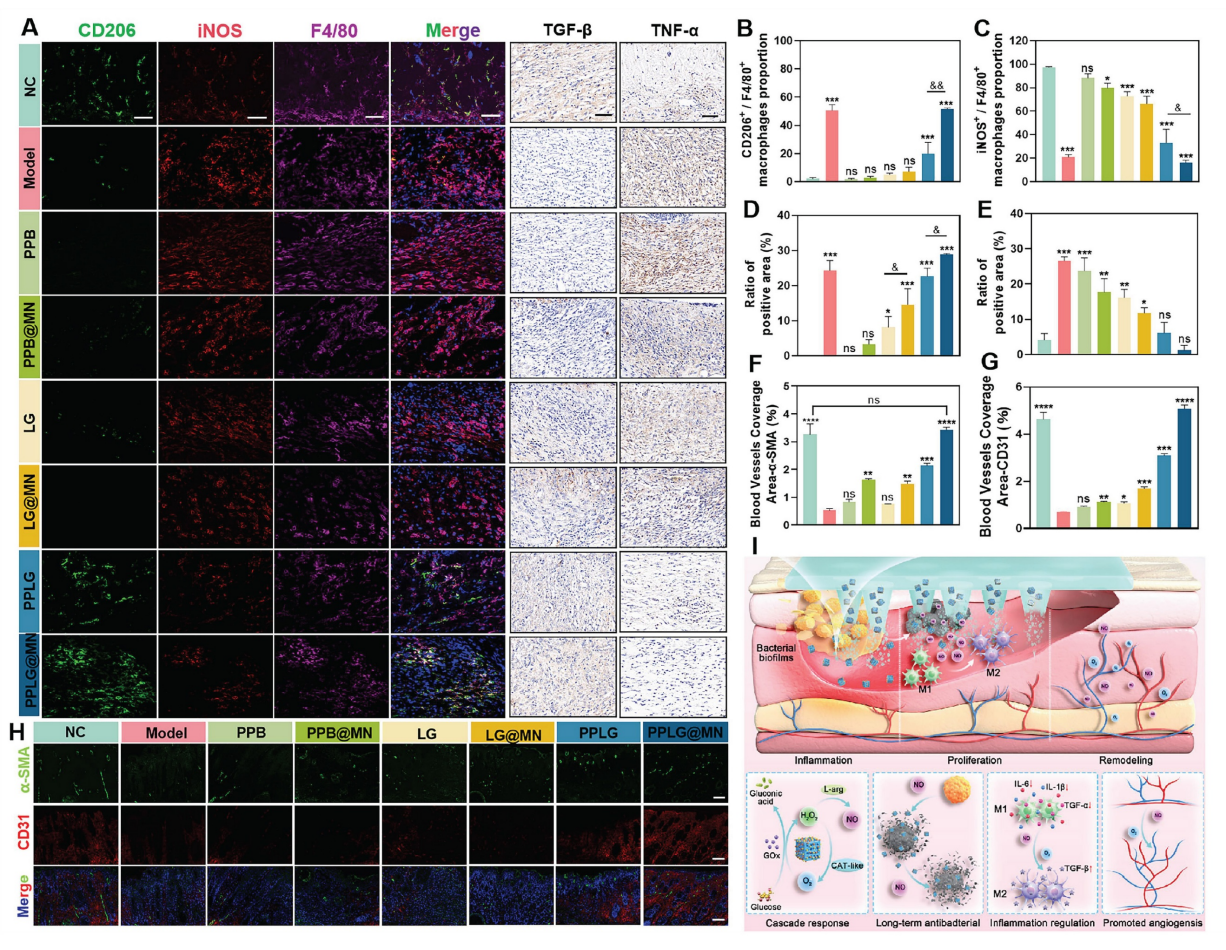
PPLG@MN promotes wound healing mechanisms *in vivo*. (A) Immunofluorescence images of F4/80 (purple), iNOS (red) and CD206 (green) in the wound sites on day 7 (scale bar: 50 μm); Immunohistochemical visualisation of TGF-β and TNF-α (scale bar: 50 μm). (B) Percentage of F4/80^+^CD206^+^ cells (n = 3) and (C) F4/80^+^iNOS^+^ cells (n = 3). (D) The epithelial regeneration rate (%) quantified to evaluate the extent of re-epithelialization in each treatment group (n = 3). (E) The collagen deposition rate (%) measured to assess extracellular matrix remodeling (n = 3). (F, G) Relative coverage area of CD31 and α-SMA in wound tissue from each group (n = 3). (H) Representative immunofluorescent staining images of CD31 (red), α-SMA (green), and DAPI (blue) in wound tissues from different groups on day 14 (scale bar: 100 μm). (I) Schematic diagram of PPLG@MN for the treatment of diabetic biofilm wounds infected with *S. aureus*. Values are expressed as the mean ± SD. '^*^' indicates *p* < 0.05, '^**^' indicates *p* < 0.01, '^***^' indicates *p* < 0.001; '^&^' indicates *p* < 0.05, '^&&^' indicates *p* < 0.01.

A prolonged inflammatory phase is a typical feature of chronic diabetic wounds, and excessive expression of inflammatory cytokines further impedes the transition of macrophages from the pro-inflammatory M1 phenotype to the anti-inflammatory M2 phenotype. To investigate the immunomodulatory effects of PPLG@MN, macrophage polarization was analyzed using quadruple immunofluorescence staining. Quantitative analysis revealed significant differences in macrophage phenotype distribution among treatment groups (Figures 6A-C). Compared with the model group, PPLG@MN treatment demonstrated: (1) a marked reduction in M1 macrophage marker iNOS (*p* < 0.01), (2) a significant enhancement of the M2 marker CD206 (*p* < 0.01), and (3) the highest M2/M1 polarization ratio among all groups. Our results demonstrate that PPLG@MN treatment significantly reduced the M1 macrophage marker iNOS while enhancing the expression of the M2 marker CD206, indicating its potential role in modulating macrophage polarization. Previous studies have shown that moderate levels of NO can regulate macrophage function by suppressing pro-inflammatory gene transcription and improving the local redox environment. In addition, increased oxygen availability can alleviate hypoxia-associated inflammatory conditions and facilitate the development of a reparative phenotype. The reductions in inflammatory cytokines and the enhanced angiogenesis observed in this study are also consistent with the characteristic immunoregulatory functions of M2 macrophages, supporting the plausibility of NO/O_2_-associated macrophage reprogramming 42, 43.

Next, to assess the degree of inflammation at the wound site, the diabetic wound samples of each group were analyzed by immunohistochemistry. The expression of pro-inflammatory cytokine TNF-α was significantly attenuated and the expression of anti-inflammatory cytokine TGF-β was significantly enhanced in the PPLG and PPLG@MN groups (Figure 6A). The quantitative results (Figure 6D-E) confirmed these findings. These results suggest that the cascade reaction promoting NO releases induces macrophages to shift an anti-inflammatory phenotype, inhibits the secretion of pro-inflammatory factors, enhances the secretion of anti-inflammatory cytokines, effectively reduces inflammation, and promotes wound healing.

Vessel formation contributes to wound healing by improving nutrient transport and oxygen supply, thereby promoting fibroblast proliferation, collagen deposition and epithelialization. To quantitatively evaluate vascularization, immunofluorescence staining was performed using the endothelial cell marker CD31 and the pericyte marker α-SMA, which reflect angiogenesis and vascular maturation, respectively. The expression levels of CD31 and α-SMA in the PPLG@MN group were much higher than those in the model group, with a particularly substantial increase in CD31 expression (Figure 6F-H). This finding suggests that vascular development in the PPLG@MN group had progressed to a more mature stage compared with the model group. These findings demonstrate that PPLG@MN promotes both angiogenesis and vascular maturation, creating a favorable microenvironment for wound repair through enhanced oxygen and nutrient supply. In addition, serum biochemical analyses and histological examinations of the heart, liver, spleen, lung, and kidney were performed in mice from the PPLG@MN and NC groups on day 14 after administration. The results showed no significant changes in aspartate aminotransferase (AST), alanine aminotransferase (ALT), blood urea nitrogen (BUN), or creatinine (Cr) levels (Figure S24), and no apparent histopathological abnormalities were observed in major organs (Figure S25). Moreover, a 24-hour dynamic assessment of serum nitric oxide metabolites (NOx, including nitrite and nitrate) revealed no abnormal accumulation, further indicating that localized NO release did not induce systemic effects (Figure S26). Collectively, these findings confirm the favorable biosafety profile of PPLG@MN.

The Figure 6I illustrates that the cascade reaction involves the consumption of excess glucose to release H_2_O_2_-NO, which collectively contributes to bacterial inhibition, inflammation reduction, and the promotion of angiogenesis. Beyond these mechanistic insights, the present system also offers key innovations compared with previously reported NO-based therapies, nanozyme platforms, and microneedle systems. Traditional NO therapies commonly rely on exogenous NO donors, which often exhibit burst release, short half-lives, and limited biofilm penetration, restricting their capacity for coordinated immune regulation 44,45. Recent nanozyme-based antibacterial systems typically focus on single-step ROS or NO generation, lacking microenvironment-responsive, self-regulated gas signaling 46,47. Meanwhile, previously reported microneedle platforms primarily enhance drug deposition but do not integrate glucose-responsive catalytic regulation. In contrast, PPLG@MN integrates glucose-driven cascade catalysis, microneedle-mediated biofilm penetration, and dynamic NO/O_2_ modulation into a unified platform capable of adapting to the evolving microenvironment of diabetic wounds. The combination of metabolic regulation, immune remodeling, and precise gas signaling distinguishes the present system from prior NO donors, nanozyme mono-catalytic systems, and non-catalytic microneedle therapies, representing a mechanistically and functionally distinct therapeutic approach.

## Conclusions

In this study, we developed a glucose-responsive cascade nanozyme microneedle system (PPLG@MN) integrating microneedle-mediated biofilm penetration, starvation therapy, and dynamic NO/O_2_ gas modulation for diabetic wound healing. Compared with conventional nitric oxide donor therapies, the PPLG@MN system is distinguished by its spatiotemporally controllable release mechanism, which precisely aligns with the dynamic changes in the wound microenvironment. This feature not only facilitates a seamless transition from antibacterial activity to pro-regenerative effects but also renders the therapeutic process more precise, controllable, and safe. PPB transcends the conventional "single catalysis" or "carrier" roles in traditional nanozyme research. It possesses the dual functionality of regulating gaseous signaling molecules and serving as a synergistic platform for two therapeutic agents, thereby driving a sustainable nitric oxide generation cycle. Crucially, the spatiotemporally controlled release mechanism of this platform precisely aligns with the dynamic wound microenvironment, facilitating a seamless transition from antimicrobial defense to tissue regeneration. In diabetic mouse models, PPLG@MN achieved a coordinated therapeutic effect through precise gas regulation, enhanced biofilm penetration, and microenvironment-responsive modulation, markedly accelerating the healing of infected wounds and promoting tissue regeneration. These results underscore the broad applicability and translational potential of this strategy. This work further demonstrates how nanozymes can mimic physiological feedback mechanisms to address the complex pathology of chronic wounds, providing a clinically meaningful approach for next-generation wound repair technologies and offering a promising solution to unmet clinical needs in the treatment of chronic diabetic wounds.

## Supplementary Material

Supplementary figures and method.

## Abbreviations

ROS: reactive oxygen species
NO: nitric oxide
PPB: Prussian blue nanozymes
GOx: glucose oxidase
L-arg: L-arginine
H_2_O_2_: hydrogen peroxide
O_2_: oxygen
CO: carbon monoxide
H_2_S: hydrogen sulfide
CAT: nanozymes exhibit catalase
MN: dissolvable microneedles
STZ: Streptozotocin
TEM: transmission electron microscopy
SEM: scanning electron microscopy
XRD: x-ray powder diffraction
XPS: x-ray photoelectron spectrometer
EDS: energy dispersive spectroscopy
CLSM: confocal laser scanning microscope
FTIR: fourier-transform infrared spectroscopy
ELISA: enzyme-linked immunosorbent assay
LG: L-arg/GOx
PPLG@MN: pplg-loaded microneedles
BF: bacterial biofilms
EPS: extracellular polymeric substance
