# Supplementary material for: Spatiotemporal NO/O2-releasing cascade nanozyme microneedles enhance diabetic infected wound healing by modulating the immune microenvironment
Source: Theranostics. 2026 Jan 1;16(6):2811–28. doi: 10.7150/thno.123827 (PMC12775821; doi:10.7150/thno.123827)
Supplement: Supplementary file 1 — Supplementary figures and method. [file thnov16p2811s1.pdf]

Supporting Information for:

Spatiotemporal NO/O_2-releasing cascade nanozyme microneedles enhance diabetic infected wound healing by modulating the immune microenvironment

Supporting Figures:

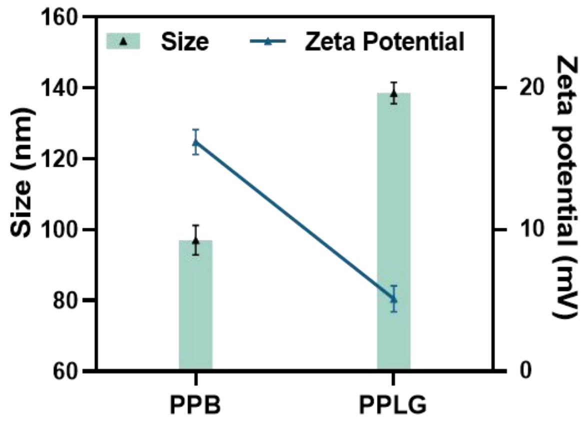

Figure S1. DLS of PPB and PPLG

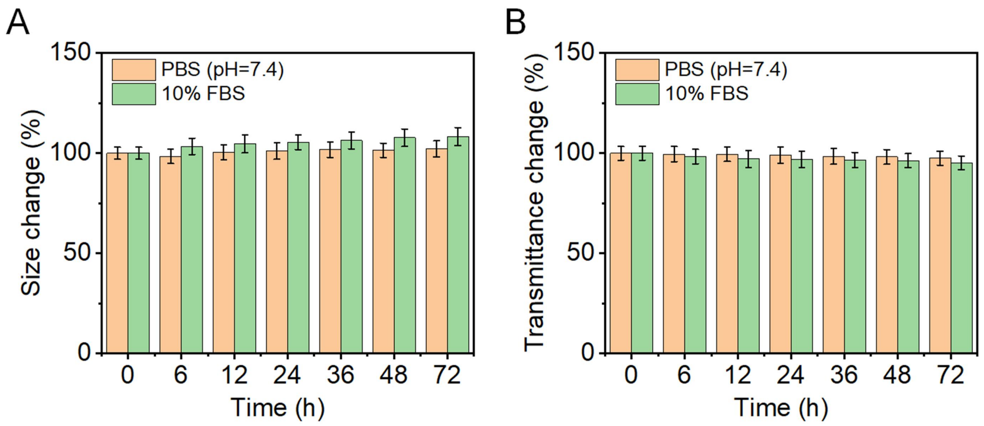

Figure S2: Particle size (A) and transmittance changes (B) of PPLG in 10% FBS and PBS

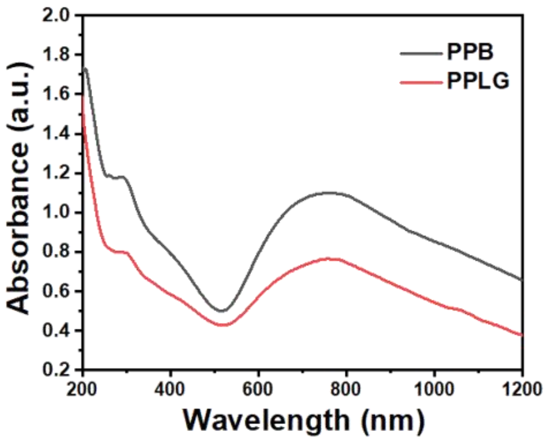

Figure S3. UV-visible spectral images of PPLG and PPB

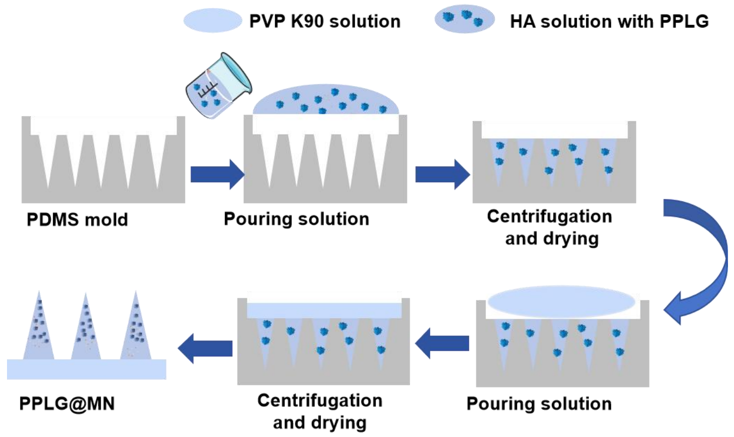

Figure S4. Schematic representation of the manufacturing process of PPLG@MN

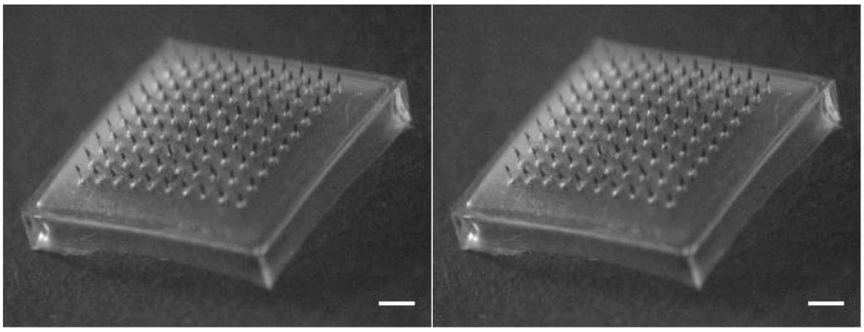

Figure S5. Digital microscope image PPLG@MN (scale bar: 1 mm)

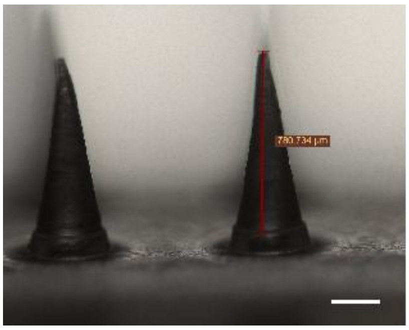

Figure S6. Light microscope image (scale bar: 200 nm)

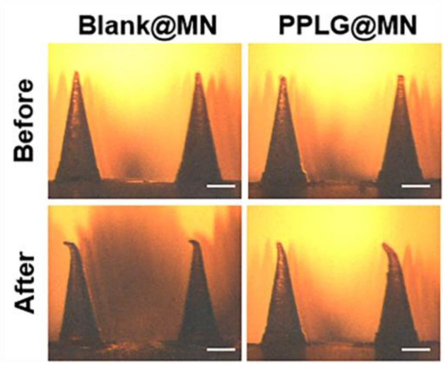

Figure S7. Mechanical strength analysis of blank MN and PPLG@MN

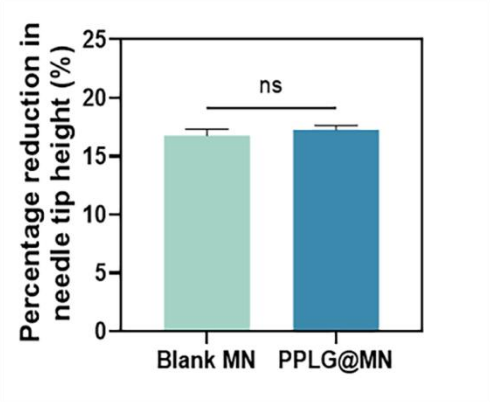

Figure S8. Quantitative analysis of mechanical strength of blank MN and PPLG@MN

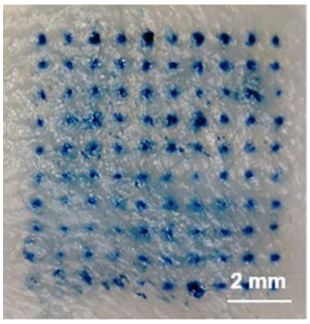

Figure S9. Image of skin stained with trypan blue (scale bar: 2 mm)

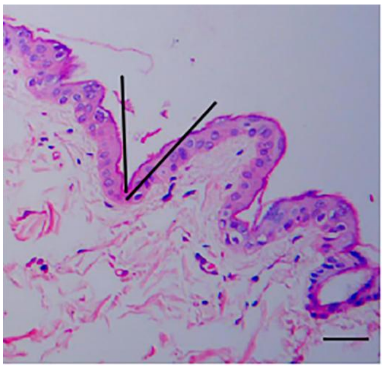

Figure S10. HE staining of skin (scale bar: 200 μm)

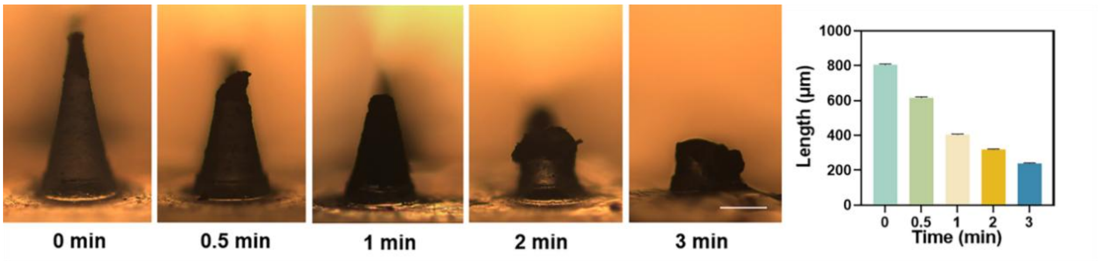

Figure S11. Quantification of the dissolution process and length change of microneedle tip (scale bar: 200 μm)

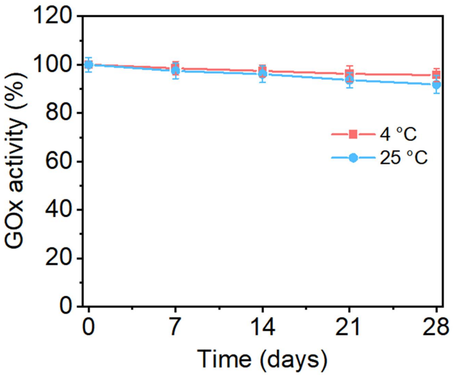

Figure S12. Long-term storage stability of GOx in the PPLG@MN system

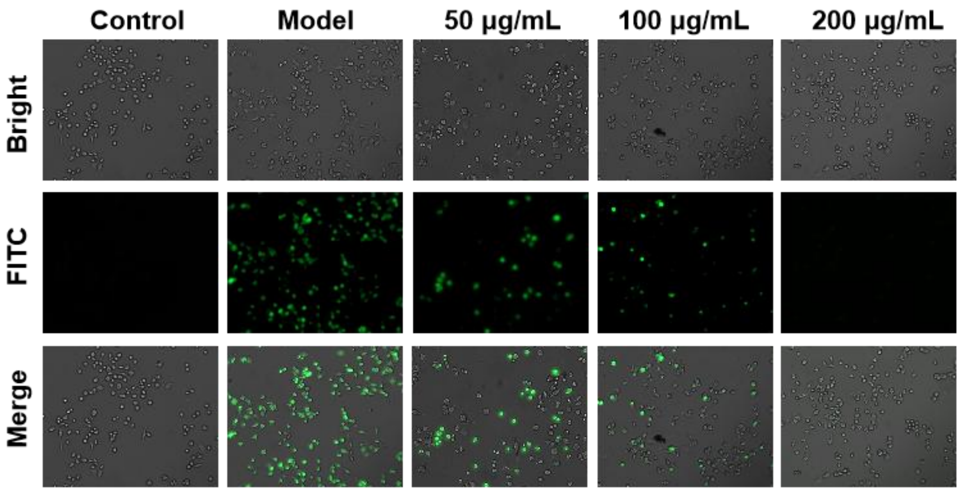

Figure S13. Scavenging efficiency of intracellular ROS by different concentrations of PPB

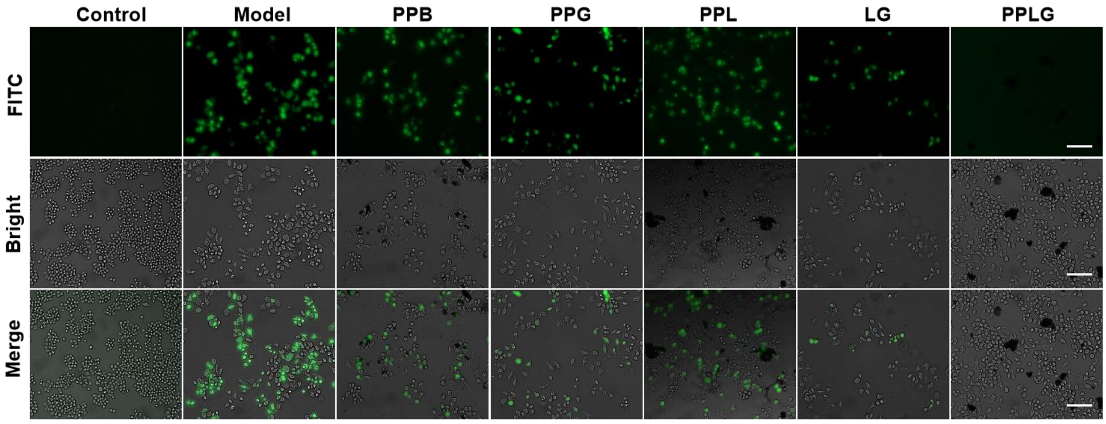

Figure S14. Fluorescence Microscopy to Evaluate ROS Scavenging Efficiency

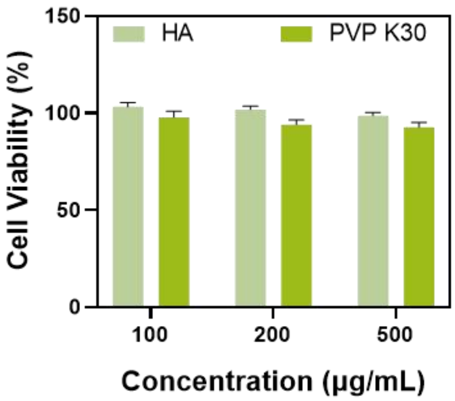

Figure S15. Biosafety of microneedle excipients

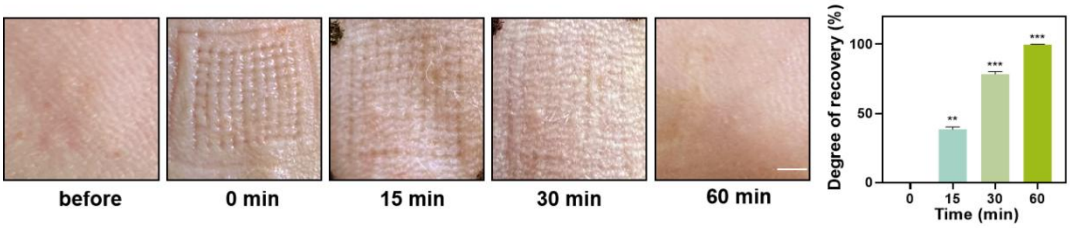

Figure S16. Skin recovery after microneedle action

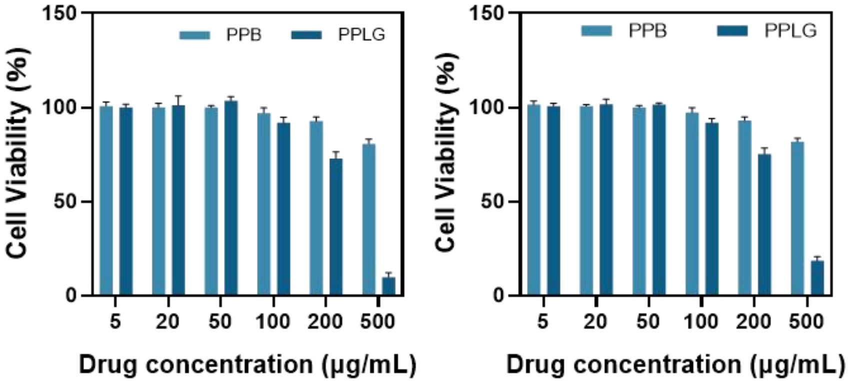

Figure S17. Cytotoxicity of different drug concentrations on HUVEC and RAW264.7 cells

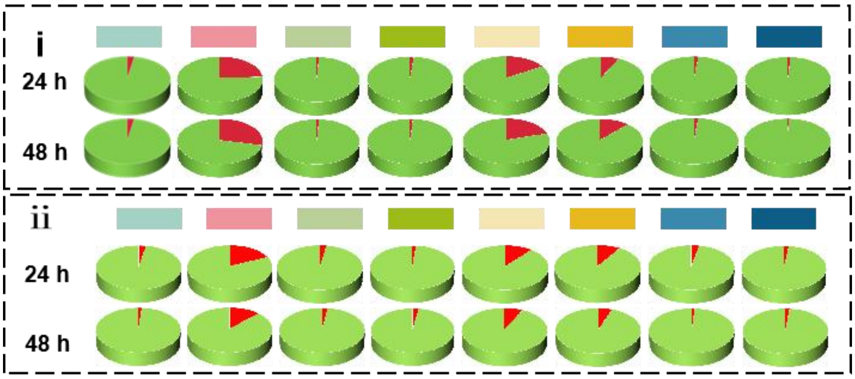

Figure S18. Live/dead cell ratios of HUVEC (i) and RAW 264.7 cells (ii) incubated for 24 h and 48 h with different samples

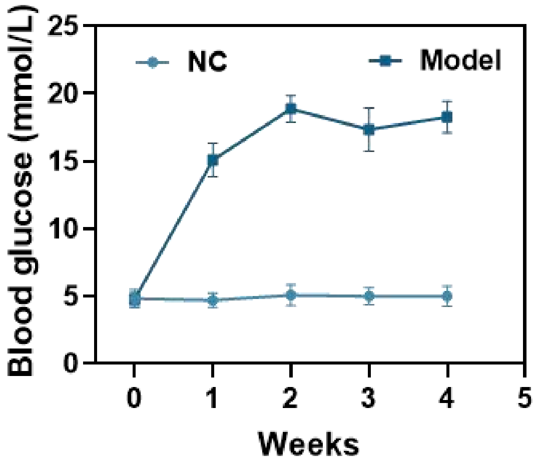

Figure S19. Blood glucose of normal and diabetic groups throughout the experiment.

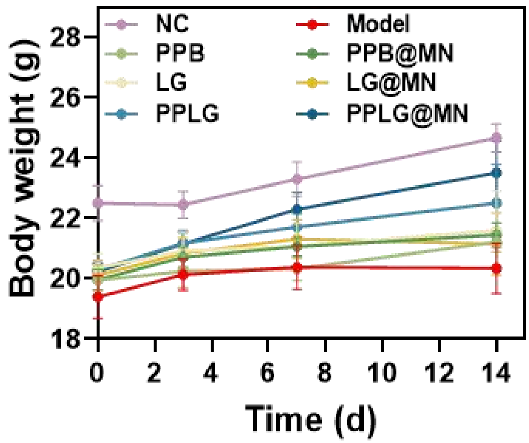

Figure S20. Body weight changes of the mice in each group during the wound healing process.

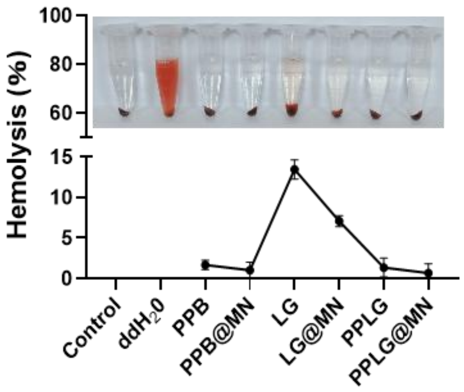

Figure S21. Haemolysis rate and optical photographs of PPLG@MN (inset)

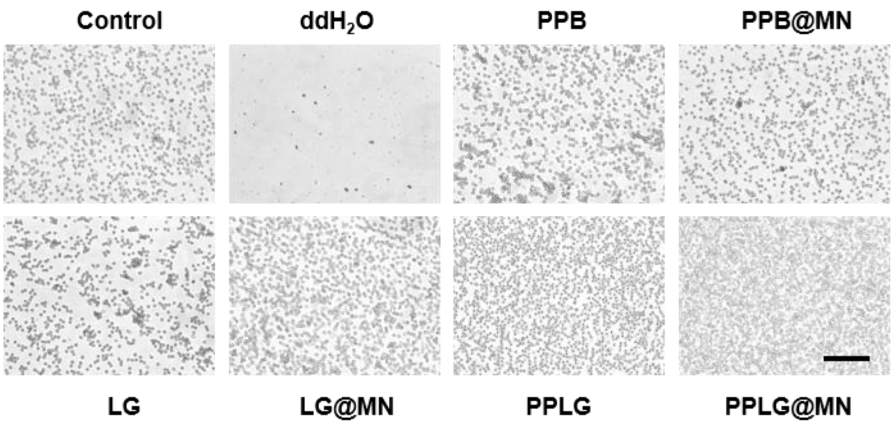

Figure S22. Morphology of erythrocytes in different groups (scale bar: 100 μm)

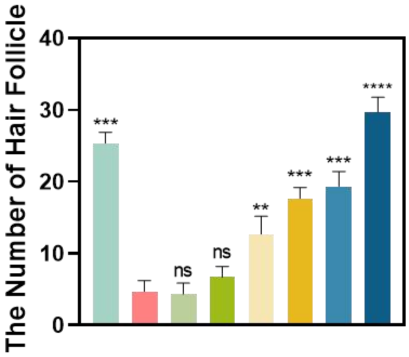

Figure S23. The number of traumatic hair follicles was determined at 14 d postoperatively

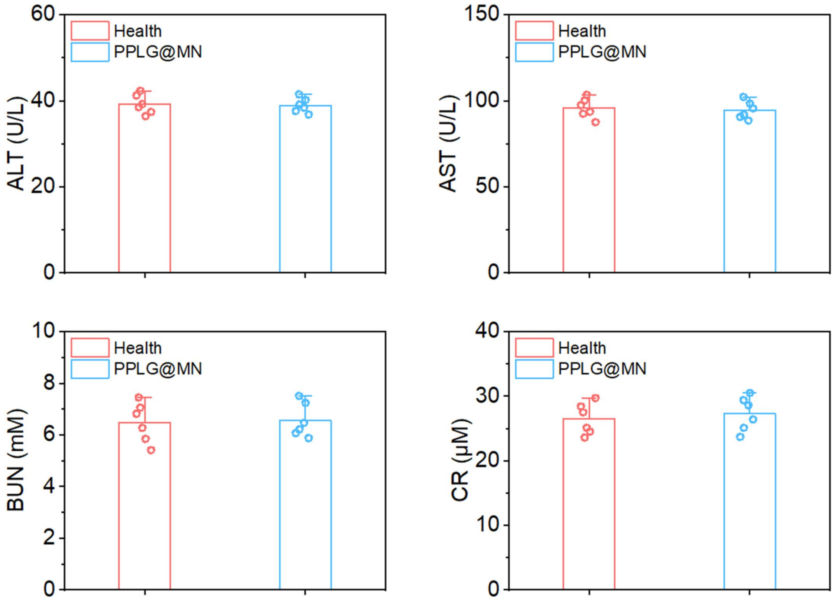

Figure S24. Serum ALT, AST, BUN, and Cr levels in healthy mice and PPLG@MN-treated mice (n=6)

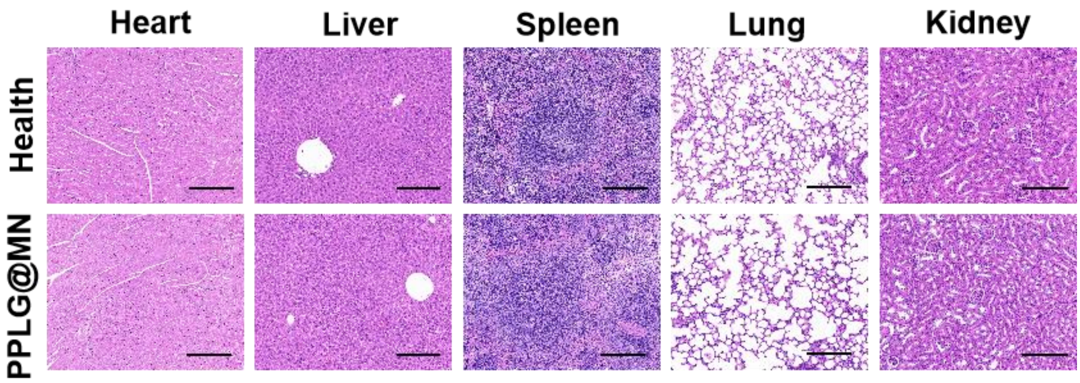

Figure S25. Histological evaluation of H&E staining of the heart, liver, spleen, lung and kidney in different treatment groups (scale bar: 200 μm)

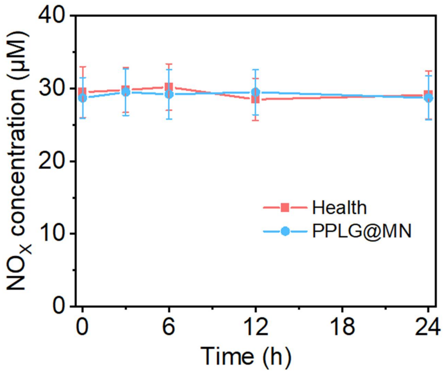

Figure S26. Time-dependent changes in serum NOx levels in healthy mice and PPLG@MN-treated mice over 24 h (n=3)

Statistical Analysis. Statistical analyses were performed using one-way or two-way ANOVA. Statistical analyses All data results are expressed as mean ± standard deviation (SD) and were performed at least three times for each experiment. 'ns' indicates non-significant ($p > 0.05$), '*' indicates $p < 0.05$, '**' indicates $p < 0.01$, '***' indicates $p < 0.001$.
